# Community Health Workers Can Identify and Manage Possible Infections in Neonates and Young Infants: MINI—A Model from Nepal

**DOI:** 10.3329/jhpn.v29i3.7873

**Published:** 2011-06

**Authors:** Sudhir Khanal, Jaganath Sharma, Vijay Singh GC, Penny Dawson, Robin Houston, Neena Khadka, Bhanu Yengden

**Affiliations:** ^1^Morang Innovative Neonatal Intervention/John Snow Inc. Research and Training Institute, Kathmandu, Nepal; ^2^Nepal Family Health Program/USAID, Kathmandu, Nepal; ^3^John Snow Inc. Research and Training Institute, Kathmandu, Nepal; ^4^Save the Children/Saving Newborn Lives, Kathmandu, Nepal; ^5^District Public Health Office, Morang, Ministry of Health and Population, Nepal

**Keywords:** Antibiotic use, Bacterial infections, Drug therapy, Health workers, Infant, Newborn, Neonatal mortality, Sepsis, Nepal

## Abstract

The mortality rates of infants and children aged less than five years are declining globally and in Nepal but less among neonates. Most deliveries occur at home without skilled attendants, and most neonates may not receive appropriate care through the existing medical systems. So, a community-based pilot programme—Morang Innovative Neonatal Intervention (MINI) programme—was implemented in Morang district of Nepal to see the feasibility of bringing the management of sick neonates closer to home. The objective of this model was to answer the question: “Can a team of female community health volunteers and paid facility-based community health workers (collectively called CHWs) within the existing heath system correctly follow a set of guidelines to identify possible severe bacterial infection in neonates and young infants and successfully deliver their treatment?” In the MINI model, the CHWs followed an algorithm to classify sick young infants with possible severe bacterial infection (PSBI). Female Community Health Volunteers (FCHVS) were trained to visit homes soon after delivery, record the birth, counsel mothers onessential newborn care, and assess the newborns for danger-signs. Infants classified as having PSBI, during this or subsequent contacts, were treated with co-trimoxazole and referred to facility-based CHWs for seven-day treatment with injection gentamicin. Additional supervisory support was provided for quality of care and intensified monitoring. Of 11,457 livebirths recorded during May 2005–April 2007, 1,526 (13.3%) episodes of PSBI were identified in young infants. Assessment of signs by the FCHVs matched that of more highly-trained facility-based CHWs in over 90% of episodes. Treatment was initiated in 90% of the PSBI episodes; 93% completed a full course of gentamicin. Case fatality in those who received treatment with gentamicin was 1.5% [95% confidence interval (CI) 1.0-2.3] compared to 5.3% (95% CI 2.6-9.7) in episodes that did not receive any treatment. Within the existing government health infrastructure, the CHWs can assess and identify possible infections in neonates and young infants and deliver appropriate treatment with antibiotics. This will result in improvement in the likelihood of survival and address one of the main causes of neonatal mortality.

## INTRODUCTION

Over the last few decades, the global trend in rates of mortality of infants and children aged less than five years (under-five children) is declining but less among neonates. The major causes of neonataldeath are infection, birth asphyxia, prematurity, and low birthweight. Most deliveries occur at home without skilled attendants, and most neonates may not receive appropriate care through the existing medical systems (1-3).

The *Lancet* series on neonatal survival noted that community-based management of pneumonia can result in a 27% reduction in all-cause neonatal mortality and classified this intervention as one with “incontrovertible efficacy, which seems feasible for large scale implementation based on effectiveness trials” (4). Community-based management of diseases in older children has been successfully tried in many countries (5). However, application of this community-based approach to address neonatal illness is limited.

A field trial in Gadchiroli, India (SEARCH), included community-based identification and treatment of suspected neonatal infection (sepsis) by trained village health workers hired by the project. The study demonstrated a 62% reduction in neonatal mortality (6). In Sylhet, Bangladesh, a similar home-care approach was used for the management of infections and showed a reduction in mortality by 34% (7). These studies were designed to assess the impact of mortality and to contribute to the management of neonatal infections but both used community health workers (CHWs) outside the existing health infrastructure.

The 2001 and 2006 Nepal Demographic and Hea-lth Surveys reported mortality for the five-year period preceding the surveys. The surveys showed a 33% reduction in under-five mortality from 91 to 61 deaths per 1,000 livebirths. Neonatal mortality declined by 15% from 39 to 33 deaths per 1,000 livebirths (8,9). Interest in maternal and child health has intensified in Nepal, resulting in a number of interventions being tested to improve maternal health and reduce risk in neonates. Some of these include preventive measures designed to reduce the risk of infection in neonates but none addresses the management of possible infection. Interventions to address birth asphyxia and prematurity/low birthweight are challenging in Nepal as skilled attendants conduct only 19% of deliveries and 18% of deliveries are conducted at health facilities (10). Due to limited contact with skilled personnel during delivery and in the immediate postnatal period, addressing treatable conditions, such as possible severe bacterial infection (PSBI) at the community level, is critical.

While reduction in mortality has been demonstrated in controlled trials, there is limited evidence on implementation of such community-based programme within the government health systems. The Morang Innovative Neonatal Intervention (MINI) is the first programmatic model in Nepalto introduce community-based identification and management of PSBI in young infants.

The main objective of the MINI was to test a replicable model for the community management of neonatal infections within the existing government health system. The specific objectives were to (a) demonstrate that Female Community Health Volunteers (FCHVs) can identify and record births, correctly assess sick neonates, initiate treatment with an appropriate oral antibiotic, and facilitate referral for injectable antibiotics for PSBI and (b) demonstrate that facility-based CHWs can respond to referrals and administer the appropriate injectable antibiotic for PSBI.

## MATERIALS AND METHODS

### National context

The Community-based Integrated Management of Childhood Illness (CB-IMCI) programme of the Ministry of Health and Population (MoHP) provides treatment only for sick children aged over two months. The MINI is expected to address this gap by including management of infants through two months of age. The MINI model was envisioned to test a sustainable and replicable approach for the MoHP to implement at scale. Hence, it was implemented through the existing government infrastructure. A specific monitoring and supervision component was added to provide adequate information on model results.

The CHWs in Nepal consist of village-based FCHVs and paid facility-based community health workers (FB-CHWs). The FCHVs are local women, with limi-ted formal education, serving voluntarily within the government system, in all the wards of Nepal. They receive basic training of 18 days and perio-dic refresher and programme-specific training. Approximately 50,000 FCHVs work actively in all the villages of Nepal and are supported by facility-based health workers.

Since 1988, the FCHVs have been given responsibility by the MoHP for health promotion. This includes supporting family-planning activities and distributing iron tablets, vitamin A, and oral rehydration solution. The FCHVs have also been trained to identify and manage pneumonia, including treatment with co-trimoxazole, in children aged over two months in over half of the country. They refer severe cases to the nearest health facili-ty (11). They reside in the communities they serve, are likely to know about pregnancies and deliveries, and are, thus, able to record births.

The FB-CHWs are the most peripheral paid government health workers. Their basic education varies from grade 8 to grade 10, and they receive in-service training, which includes injection skills. They routinely manage immunization outreach clinics.

### Setting

Morang district, located in the eastern part of Nepal, was selected as the site for the MINI. Morang has primarily flat agricultural land, with some adjoining hills, with an area of 1,855 sq km. It has a population of 9,14,799, 80% of whom live in rural areas (12). The human development and other indices of Morang (13,14) compared to the national figures indicate no major differences (Table 1).

**Table 1.** Human development and other indices

Indicator	National	Morang
Human development index (13)	0.471	0.531
Human poverty index (13)	39.6	34.4
Per-capita income (US$) (13)	240	297
Literacy rate—6 years and above (14)	48.6	57
Male	62.7	67.1
Female	34.9	46.8

Districts in Nepal are divided into administrative and political units, called Village Development Committees (VDC), each of which is divided into nine wards. Municipalities were not included in the model. Twenty-one of the 65 VDCs of Morang, with a total population of 2,98,588, were selected for the MINI model. The selection was based on comparability with the remaining VDCs of the district, with respect to proximity to secondary health facilities and other community characteristics. Each VDC has one government health facility and nine FCHVs, i.e. one per ward, working in their own community. Each FCHV serves an average population of 1,580, and each health facility, which has at least three FB-CHWs, serves a population of 14,219 in the catchment area.

According to the profile data on district health workers, 81% of 189 FCHVs are literate, of whom 60% studied beyond the primary level. Pictorial and colour-coded training materials and forms were used for training the FCHVs and for the recording and reporting purposes.

### Study design

A Technical Working Group (TWG) was established to guide all technical aspects of implementation of MINI. The TWG and national neonatal experts approved the clinical algorithm for PSBI based on those used in SEARCH/India and the WHO's Young Infant Studies (15,16). The final MINI algorithm was compared with other algorithms in Table 2. The TWG also approved the antibiotic regimen for PSBI based on the modified WHO guidelines: paediatric oral co-trimoxazole tablets (crushed and dissolved in breastmilk) twice daily for five days and intramuscular gentamicin injections (10 mg for infants of <2.5 kg and 15 mg for infants of >2.5 kg) once daily for seven days (17).

**Table 2.** Comparison of signs in MINI algorithm with other algorithms for possible infection

MINI algorithm	WHO algorithm	SEARCH algorithm
Presence of any one of the following signs	Presence of any one of the following signs	Presence of any two of the following signs on same day
Unable to feed	Convulsions	Reduced feeding or stopped feeding
Lethargic or unconscious	Lethargic or unconscious	Drowsy or unconscious
Fast breathing (≥60 breaths per minute)	Fast breathing (60 breaths perminute or more)	
Severe chest in-drawing	Severe chest in-drawing	Chest in-drawing
Grunting	Grunting	
Fever (temperature ≥37.5 °C)	Fever (37.5 °C* or above or feels hot)	Mother says baby becomes cold to touch (hypothermic)
Hypothermia (temperature <35.5 °C)	Low body-temperature (lessthan 35.5 °C* or feels cold)	Or temperature is more than 99 ^o^F (37.2 °C)
Redness around umbilicus	Umbilical redness extending to the skin or	Pus in umbilicus
>10 skin pustules or 1 large abscess	Many or severe skin pustules	Or abscess with pus in skin
Weak cry		Weak cry or stopped cry
	Nasal flaring	
	Bulging fontanelle	
	Pus draining from ear	
	Less than normal movement	
		Stomach distended or repeated vomiting

MINI=Morang Innovative Neonatal Intervention;

WHO=World Health Organization;

SEARCH=Society for Education, Action and Research in Community Health

The FCHVs have antenatal contact with pregnant women to provide them with iron folate tablets and advice on antenatal care. The MINI trained FCHVs to conduct a postnatal visit for all births in their wards, ideally within 24 hours of delivery, after being notified of the birth. The FCHV weighs the neonate, records the birth, encourages the mother to register the birth with the VDC, and counsels on essential newborn care (ENC). She educates the family about danger-signs for the newborn and the mother and advises when to call her. Finally, she assesses the baby for signs of possible infection. If any signs are present, she initiates treatment and referral.

If the young infant is sick at any time within the first two months, a family member calls the FCHV to examine the baby for danger-signs. If any danger-sign is present, she classifies the illness as PSBI. With consent of caretakers, the FCHV assists them in giving the first dose of paediatric oral co-trimoxazole tablets and provides the family with tablets to be given twice daily for five days. After giving the first dose of antibiotic, she sends a family member of the sick baby to call the FB-CHW who is trained and qualified to give intramuscular gentamicin injections. The FCHV subsequently makes a third-day follow-up visit.

After responding to the call and obtaining oral consent for treatment from the guardian, the FB-CHW gives gentamicin injections once daily for seven days. The location where the injection is provided varies between the home and the health facility, depending on the local situation and discussion with the family. All treatments were given free of charge.

**Fig. 1.**
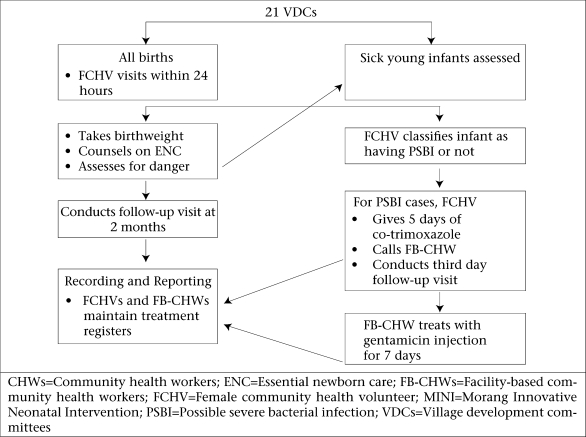
Flow-chart of MINI approach

All babies whose births were recorded during the study period were enrolled as participants in the MINI. The FCHV makes a two-month follow-up visit to all identified newborns to determine the child's survival status and the outcome of any treatment. All FCHV and FB-CHW services are recorded in registers designed specifically for the MINI, thus creating a longitudinal database for all recorded births. Figure 1 provides a flow-chart of the MINI intervention.

The MINI team and the District Public Health Office (DPHO) developed training, communication and recording materials. Taking into consideration the semi-literate status of many FCHVs, pictorial materials were drafted, pre tested, and revised before finalization. In total, 189 FCHVs and 83 FB-CHWs were trained on the assessment and management of neonatal infections and ENC messages. Training manuals, registers, reporting forms, and drugs were provided to all health workers through the existing government system. Allowances for training were paid to them as per the government rules and regulations but no cash incentives were provided for service-delivery.

Regular supervision for programme implementation was provided through the existing government system. In addition, five MINI field supervisors provided support to reinforce clinical skills of FCHVs and FB-CHWs and to collect specific data as part of the expanded monitoring system of the model.

### Source of data and measurements ofquality of data

Training began in February 2005 and was completed in early May 2005. The longitudinal database used in this paper covers the May 2005–April 2007 period. Descriptive analysis of these data is presented below.

The FCHVs and FB-CHWs used pictorial service registers specifically developed to record all data relating to the MINI intervention. The illiterate FCHVs were capable of using these pictorial registers with family support for minimal written information. Specific forms were designed to collect information from the FCHV and CHW registers, and these data were consolidated by the MINI supervisors. Coreindicators were derived from these registers. Data were entered into the MINI database using the Microsoft Access software and cleaned. The quality of data was maintained using field verification of a random selection of records and a 1:10 double entry data check. Data were analyzed using the SPSS software (version 15.0) for Windows.

A household survey was used for collecting information about healthcare-seeking behaviour of clients and adverse events. Quarterly interviews with families and caretakers were completed in almost 10% of cases to verify information in the service registers.

### Ethical issues

The study was conducted in full accordance with the ethical principles and free and informed verbal consent of the subjects or their legal guardians. The MoHP approved the investigation. Ethical approval to conduct the study was obtained from the Western Institutional Review Board in the USA.

## RESULTS

The study area had a total population of 298,588, with 11,436 expected pregnancies and 10,555 expected livebirths in 2005-2006. During May 2005–April 2007, the FCHVs recorded 11,457 livebirths, giving a crude birth-capture rate of 54%. However, other programmes have questioned the accuracy of census figures and population projections in light of the rapid decline in the total fertility rate (TFR) (9). Using a correction factor based on actual population counts done in a similar district by the Nepal Nutrition Intervention Project—Sarlahi (NNIPS)—a Johns Hopkins University research study, the birth capture rate was 74% (Tielsch J. Personal communication, 2007). The FCHVs visited 63% of babies within seven days of birth. They completed a two-month follow-up visit for 97% of births identified.

There was some variation in which signs were observed by the FCHVs and FB-CHWs perhaps relating to the difference in timing of assessment. However, the FCHV's classification of a neonate as having PSBI matched the classification of the more highly-trained FB-CHW in all episodes. Among assessments of sick neonates done on the same day, the matching by algorithm sign is shown in Table 3. The overall agreement, i.e. agreement for both presence and absence of the sign, was greater than 75% (Kappa >0.75) for all signs.

**Table 3.** Comparison of FCHV and FB-CHW assessment of PSBI signs on the same day (n=653)

Sign	Kappa statistic	95% CI
>10 skin pustules or 1 large abscess	0.96	0.90-0.98
Fever	0.85	0.77-0.91
Hypothermia	0.84	0.76-0.90
Redness around umbilicus	0.84	0.76-0.90
Unable to feed	0.82	0.73-0.88
Grunting	0.82	0.73-0.88
Fast breathing	0.80	0.71-0.87
Severe chest in-drawing	0.77	0.68-0.84
Lethargic or unconscious	0.74	0.65-0.82
Weak cry	0.71	0.62-0.79

CI=Confidence interval;

FB-CHWs=Facility-based community health workers;

FCHV=Female community health volunteers;

PSBI=Possible severe bacterial infection

**Fig. 2.**
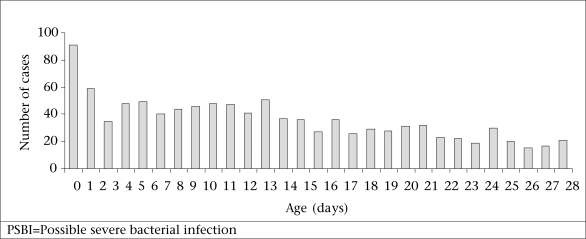
Age distribution of PSBI episodes in neonates (n=1,048), May 2005-April 2007

During May 2005–April 2007, of the 11,457 recorded births, 1,526 episodes of PSBI were recorded in 1,448 young infants. Five percent of sick young infants had more than one episode of PSBI. The overall incidence of PSBI was 13% in infants aged 0-60 days. There were 1,051 (9%) episodes of PSBI among neonates aged 0-28 days. The age distribution for PSBI episodes in neonates is shown in Figure 2. The distribution shows that the episodes of PSBI continue to occur throughout the neonatal and young infant period.

**Fig. 3.**
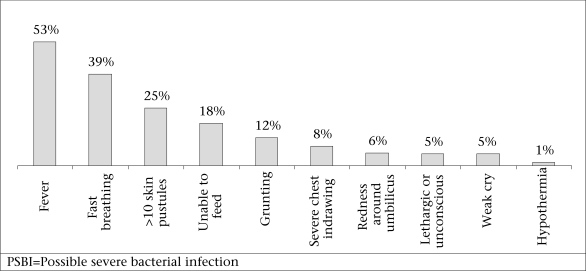
Distribution of PSBI signs in young infants (n=1,526 episodes), May 2005-April 2007

The distribution of signs of the 1,526 episodes is presented in Figure 3. Fifty-two percent of the PSBI episodes had two or more signs. The three most common signs were fever (53%), fast breathing (39%), and greater than 10 skin pustules (25%).

Of the 1,526 PSBI episodes identified, 1,376 (90%) had treatment initiated with co-trimoxazole, followed by gentamicin. Of those who initiated treatment with gentamicin, 1,279 (93%) completed the full seven-day course. Of the 150 episodes that did not receive treatment, the family did not consent with treatment for 43 episodes, and for another 21 episodes, the family did not initiate treatment due to difficulties with the FB-CHW. Twenty-one episodes were referred to a higher-level facility from which follow-up data were not available. The status of another 45 episodes was not known because they never came in contact with the FB-CHW. For the remaining 20 episodes, treatment was not received because of family issues (e.g. the caretaker had to travel to her maternal home outside the catchment area). Less than 2% (21 of 1,526) of the episodes had problems with the FB-CHW.

Before the initiation of the MINI, a few sick young infants were recorded as being treated at health facilities, and little was known about their care. Only 3% of expected PSBI cases were recorded as seen at the health facilities based on an incidence of PSBI of 10.5% (n=6) of expected births.

The timing of treatment for PSBI is important, and the MINI attempted to ensure early treatment. During the intervention period, 75% of the PSBI episodes, identified by the FCHVs or FB-CHWs, had gentamicin injection initiated within two days of identification.

During the course of the intervention, no adverse events were brought to the attention of FCHVs, FB-CHWs, or MINI staff. In a household survey, mothers of sick babies treated with gentamicin injection were asked if they noticed any adverse event. Of the 620 mothers interviewed, 73 (12%) had young infants treated. Of these, 14 (19%) reported redness around the injection site.

**Fig. 4.**
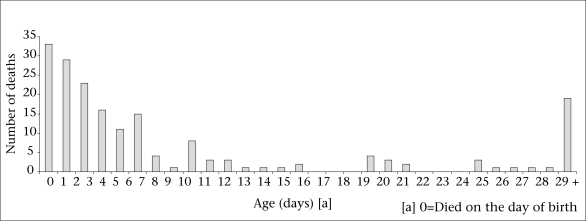
Age distribution of deaths (n=186), May 200-April 2007

During May 2005–April 2007, the MINI recorded 186 deaths. Figure 4 shows the age distribution of these deaths. Of these deaths, 29 (16%) were identified with PSBI. Of the 29 who died with PSBI, four received no treatment, 21 did not complete treatment, and three of the four who completed treatment died more than seven days after the completion of treatment. Table 4 shows the case-fatality rates for the treated and untreated PSBI episodes. Case fatality was significantly lower in the treated cases. Table 5 breaks down case fatality according to the number of algorithm signs. Case fatality by timing of initiation of gentamicin is shown in Table 6. There was no statistical difference in case fatality by timing of treatment.

**Table 4.** Case fatality rate among PSBI episodes in young infants

Characteristics	No. of episodes	No. of deaths (n=186)	MINI case fatality (%)and 95% CI
Those who received treatment	1,376	21	1.5 (1.0-2.3)
Those who did not receive treatment	150	8	5.3 (2.6-9.7)
Total PSBI episodes	1,526	29	

CI=Confidence interval;

PSBI=Possible severe bacterial infection

**Table 5.** Case fatality in PSBI episodes: comparison between one versus two signs

Morbidity category	No. ofepisodes	No. of deaths	MINI case fatality (%)(95% CI)
PSBI episodes with single sign	738	5	0.7 (0.3-1.6)
Those who received treatment	661	3	0.5 (0.2-1.3)
Those who did not receive treatment	77	2	2.6 (0.7-9.0)
PSBI episodes with two or more signs	788	24	3 (2.1-4.5)
Those who received treatment	715	18	2.5 (1.6-3.9)
Those who did not receive treatment	73	6	8.2 (3.8-16.8)
Total PSBI episodes	1,526	29	

**Table 6.** Case fatality by duration of initiation of gentamicin injection from identification of PSBI (n=1,376)

Day of treatment initiation	No. of episodes	No. of deaths	Case fatality (%)	95% CI
Same day	572	12	2.1	1.2-3.6
Day 1	321	3	0.9	0.3-2.7
Day 2	246	5	2.0	0.9-4.7
Day 3	138	0	0	
Day 4	48	1	2.08	0.4-10.9
Day 5	31	0	0	
Day 6 or more	20	0	0	
Total	1,376	21	1.5	1-2.3

CI=Confidence interval;

PSBI=Possible severe bacterial infection

## DISCUSSION

The MINI model demonstrated that the CHWs, within the existing government system, can coordinate with their community members to be notified of deliveries, classify those infants who become sick, and initiate appropriate, safe and timely care. At the community level, the FCHVs with limited education can effectively record births and also increase the likelihood of appropriate treatment for young infants who become ill. The data suggest that this community-based identification and management of PSBI can reduce the likelihood of death. The MINI provides the MoHP with a model that can be replicated in other districts. The experience in Morang could be replicated in other country settings but would depend on a strong, well-supported cadre of community-based workers.

The FCHVs recorded a high proportion of expected births, and discussions with the FCHVs by project-hired supervisors during supervisory visits suggest an even higher proportion. However, calculation of expected births is difficult because the projected census data may not reflect the true population. Nepal has experienced a dramatic decline in fertili-ty (9), and this has likely made the projected population and percentage used for calculating expected pregnancies higher than actual pregnancies. To adequately assess the true birth-capture rate, a new census is needed.

The algorithm identifies PSBI cases with good agreement between the FCHVs and the FB-CHWs. However, there is currently no gold standard against which to compare the MINI algorithm. Therefore, it is possible that the algorithm overestimates the incidence of PSBI. However, to avoid missing PSBI cases, a certain percentage of over-diagnosis is acceptable but the ideal percentage is unknown.

The model demonstrated that PSBI cases can be effectively managed by CHWs, with achievement of high rates of treatment. This was somewhat unexpected, given the difficulty in mobilizing health facility-based workers for a daily contact with individual infants. However, these rates were achieved through a process of community-level discussion about the place of treatment. Ninety percent ofshe identified PSBI episodes complied with referral advice. In Bangladesh, referral compliance is 54% (18). Comparison is difficult, however, because, in the MINI, the referral was accompanied by initiation of oral treatment, and the FB-CHWs often came to the newborn's home to give gentamicin. Whereas, in Bangladesh, referral consisted of only a recommendation to visit a higher-level facility.

The MINI increased the likelihood that a young infant with PSBI would receive appropriate treatment through the government system. The percentage of expected cases recorded as seen at the health facilities increased from 3% to 25%, and the FCHVs saw an additional 43% of expected cases. So, 68% of the expected PSBI cases were managed through the programme. The expected cases were calculated based on the findings from similar studies (6).

Treatment was immediately initiated once the CHWs came in contact with the cases. However, the period of time from the onset of illness to seeking treatment depends on the caretaker's awareness and behaviour. The MINI does counsel caretakers for early care-seeking.

The model suggests a reduction in case fatality with initiation of treatment. Case fatality as found in the MINI was lower than that found in the SEARCH study (6). In SEARCH, the case-fatality rate among treated cases (6.9%; 95% CI 4.9-9.7) was higher, possibly due to active case detection and more rigorous diagnostic criteria with higher specificity. Both the studies showed a significant reduction in case fatality among the treated cases.

### Limitations

Some births may have gone unrecorded if the infant died within the first few hours of birth or if the mother delivered outside the catchment area and/or stayed outside the area for more than two months after delivery. These life-events remain invisible to this model. The birth-capture rate in the MINI was low (54%) compared to the SEARCH study (nearly 90%). The MINI calculation was based on expected pregnancies from the national census projections while the SEARCH study actively registered pregnancies.

Some PSBI cases were likely to be missed because there was no active case detection, and the mothers might not notify the FCHV when their children were sick. There are currently inadequate data with which to estimate the true incidence of PSBI. The incidence in the MINI area compared favourably with that reported in other settings (19). It is possible that the MINI diagnostic algorithm resulted in some false positives, increasing the apparent incidence of PSBI.

While the model demonstrated good efficacy of the drug regimen used among the treated cases, the antibiotic resistance pattern was not assessed. Long-term adverse events from treatment with gentamicin cannot be tracked within the existing system. However, results of pharmacodynamic studies suggest a limited risk from gentamicin given by this regimen (20).

The MINI has a strong monitoring system that includes error checking, and virtually all cases seen by the FCHVs are recorded.

This model is successful in the *terai* or flat land of Nepal. Replication of this model in more geographically-challenging areas may make it difficult to ensure rapid treatment, and modification may be needed.

The MINI was not designed to capture data on mortality, and it is possible that the MINI missed early deaths and sick young infants who died very rapidly. This may affect the estimates of case fatality.

Hypothermia might have been underestimated because of the type of thermometer used which is not designed to capture hypothermia.

### Conclusions

The implications from the MINI are far-reaching. The results suggest that one of the major causes of neonatal mortality can be effectively managed using the existing cadre of CHWs who do not have to be highly educated. Using a simple algorithm for identifying PSBI, focused training, regular supervisory support, and improved coordination between the community and the facility-based workers, an increased number of infants can receive treatment for a potentially life-threatening infection. This highlights that the interaction between the families and the CHWs, initiated during an early home-visit after birth, increases the likelihood of survival of the baby through early identification and appropriate management of infection, even without active case detection.

## ACKNOWLEDGEMENTS

Funding was provided by the Bill & Melinda Gates Foundation through Saving Newborn Lives/Save the Children-USA to the JSI Inc. Research and Training Institute. Additional support was provided by the United States Agency for International Development, Nepal, through the Nepal Family Health Program/JSI R&T. The authors have not entered into any agreement with the funding agencies that may have limited their ability to complete the research and have had full control over the primary data. The authors thank Dr. Stephen Hodgins (NFHP/USAID/JSI/Nepal), Dr. B.D. Chataut (Nepal), Dr. Steve LeClerq (NNIPS/JHU/Nepal), Dr. Shyam Thapa and Dr. Steve Wall (SC/SNL), and Dr. Penelope Riseborough, Mary Carnell, and Kate Beal (JSI) fortheir review of the earlier version of this paper. They also thank the entire team in the District Public Health Office for their continuous support and vigilance over the programme. Sincere thanks are extended to Mr. Dinesh Neupane, Statistician, for helping with the rigorous review of the database for the revision of the manuscript. The opinions expressed herein are those of the authors and do not necessarily reflect the views of any concerned agency.
